# Influence of Pd-Layer Thickness on Bonding Reliability of Pd-Coated Cu Wire

**DOI:** 10.3390/mi15070931

**Published:** 2024-07-22

**Authors:** Junling Fan, Donglin Yuan, Juan Du, Tao Hou, Furong Wang, Jun Cao, Xuemei Yang, Yuemin Zhang

**Affiliations:** 1 School of Chemical and Environmental Engineering, Jiaozuo University, Jiaozuo 454000, China; hgxybgs@126.com (D.Y.); juandj@126.com (J.D.); houtao@jzu.edu.cn (T.H.); yxm01@126.com (X.Y.); 2 School of Mechanical and Power Engineering, Henan Polytechnic University, Jiaozuo 454000, China; wangfr@hpu.edu.cn (F.W.); cavan@hpu.edu.cn (J.C.); zhangyuemin@hpu.edu.cn (Y.Z.)

**Keywords:** Pd-coated Cu wire, Pd-layer thickness, reliability

## Abstract

In this paper, three Pd-coated Cu (PCC) wires with different Pd-layer thicknesses were used to make bonding samples, and the influence of Pd-layer thickness on the reliability of bonded points before and after a high-temperature storage test was studied. The results show that smaller bonding pressure and ultrasonic power lead to insufficient plastic deformation of the ball-bonded point, which also leads to small contact area with the pad and low bonding strength. Excessive bonding pressure and ultrasonic power will lead to ‘scratch’ on the surface of the pad and large-scale Ag spatter. The wedge-bonded point has a narrowed width when the bonding pressure and ultrasonic power are too small, and the tail edge will be cocked, resulting in false bonding and low strength. When the bonding pressure or ultrasonic power is too large, it will cause stress concentration, and the pad will appear as an ‘internal injury’, which will improve the failure probability; a high-temperature environment can make Cu-Ag intermetallic compounds (IMCs) grow and improve the bonding strength. With the extension of high-temperature storage time, the shear force of Pd100 gradually reaches the peak and then decreases, due to Kirkendall pores caused by excessive growth of IMCs, while the shear force of Pd120 continued to increase due to the slow growth rate of IMCs. In the high-temperature storage test, the thicker the Pd layer of the bonding wire, the higher the bonding strength; in the cold/hot cycle test, the sample with the largest Pd-layer thickness has the lowest failure rate. The thicker the Pd layer, the stronger its ability to resist changes in the external environment, and the higher its stability and reliability.

## 1. Introduction

Wire bonding is a key method of making electrical interconnections between integrated circuit chips and lead frames in microelectronics [1,2,3,4]. However, Cu has better electrical performance; its conductivity is twice as high as gold, and the cost is five-to-ten times less. Thus, the Cu-wire bonding remains a compelling choice in microelectronic packaging [5,6,7]. Cu-wire bonding will be a potential alternative interconnection technology [8,9].

The microstructure of the bonding interface is associated with the bonding performance [10]. The bonding strength and reliability are mainly determined by the IMCs at the bonding interface. The formation of an appropriate amount of IMCs at the bonding interface will increase the bonding strength, but too many or too few IMCs may lead to the decline in bonding performance [11,12,13]. Reliability in electronic packaging refers to the ability of the product to complete the specified functions under the specified conditions and within the specified time. The reliability test refers to accelerating the reaction of the service status of the product in the actual use environment, verifying and obtaining the quality status of the product by simulating the changes in temperature, humidity and force in the real environment [14]. The mechanical reliability of wire bonding in microelectronic packaging largely depends on the formation and growth of IMCs between the bonding point and pad, so IMCs are necessary for successful bonding [15,16]. The market mainly uses Al-based coatings for LED substrates, while Xu et al. [17] utilized a Ag-coated layer as the surface material of the packaging substrate, increasing the forward light output efficiency through its mirror-like reflective effect. Li [18] used Cu-coated Ag nanoparticles to perform bonding tests, and due to the excellent low-temperature sintering properties of Ag, the bonding interface was highly compact and almost defect-free. As the Ag content increased, a more compact bonding interface was achieved, with almost no visible defects or pores. Tian et al. [19] demonstrated that the broader fusion brought about by Ag nanoparticles could create a more stable interconnection between Cu and Ag.

Scholars have conducted in-depth studies on the growth rate of IMCs of PCC and the effect of Pd on the growth rate of IMCs. Early studies have shown that the IMCs of bare Cu wire are hard and brittle [20,21,22], but in the IMCs of PCC wire, due to the small difference in electrical properties between Pd and Cu [23,24], it is easy to form a solid solution with a face-centered cubic structure, so the IMCs of PCC wire have better ductility and slower growth rate [25]. It is reported that IMCs in PCC wire bonding are thinner than bare Cu-wire bonding [26], because Pd can slow down the growth rate of IMCs in PCC wire bonding [27,28,29,30,31]. Xu et al. [32] compared the IMC thickness between the bonding interface of bare Cu wire and PCC wire at the aging temperature of 175 °C; due to the re-diffusion and accumulation of Pd in the peripheral area of the bonding interface during the aging process, Pd in the central area is very limited. Therefore, the thickness of IMCs in the peripheral region of PCC wire bonding interface becomes thinner with the increase in time, and the thickness of IMCs in the central region is similar to that of bare Cu wire. Some scholars found that the reason why Pd can slow down the growth rate of IMCs is that the Pd-rich layer at the bonding interface acts as a diffusion barrier between atoms [29,33,34,35]. In addition, research shows that Pd at the bonding interface helps to maintain the bonding strength after the aging test [36].

However, most of the above studies focused on the IMC characteristics of PCC, but little on the evolution of Cu-Ag IMCs in a high-temperature environment. In this paper, the characteristics of IMCs between PCC and the Ag pad and the function of Pd in IMCs were studied through a high-temperature storage test and cold/hot cycling test. The influence mechanism of IMCs on bonding strength and reliability was explained, which contributed to the application of PCC.

## 2. Experimental Process

**Figure 1:**
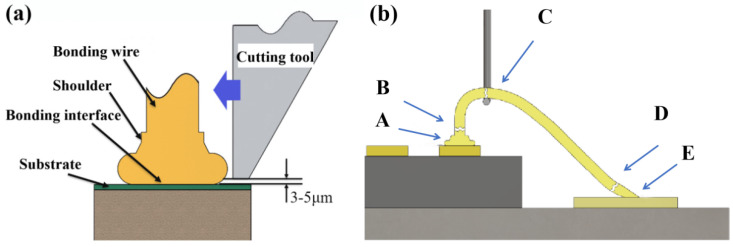
Ball-thrust/wire-breaking force test. ((**a**) Schematic diagram of shear force test of ball-bonded point; (**b**) schematic diagram of wire-breaking force test.)

Tests were conducted using three Pd-layer thicknesses (80 nm, 100 nm, and 120 nm) of PCC bonding wires with a copper matrix diameter of 25 μm, and bonding wires numbered Pd80, Pd100, and Pd120, respectively. Three types of PCC were subjected to bonding tests on LED chips using a KAIJO FB-988 bonding machine, and the optimal parameter range of bonding pressure and ultrasonic power was determined by an orthogonal test. The morphology of the ball-bonded point and wedge-bonded point were observed by Quanta EFG 250 field-emission scanning electron microscope (SEM). The breaking force of the bonding wire and the shear force of the ball-bonded point were tested on the push–pull tester, as shown in Figure 1. The schematic diagram of the shear force of the ball-bonded point is shown in Figure 1a. In general, there are only five failure positions in the bonding-wire tension test, as shown in Figure 1b. Point A is the ball-bonded interface, which can reflect the strength of the ball-bonded point; point B is located in the heat affected zone, reflecting the strength of the neck of the ball-bonded point; point C is the tensile testing point, which reflects the strength of the bonding wire itself; point D is the front end of the wedge-bonded point, with a relatively small amount of deformation; and the E point is the fish-tail part, which reflects the strength of the wedge-bonded point.

**Figure 2:**
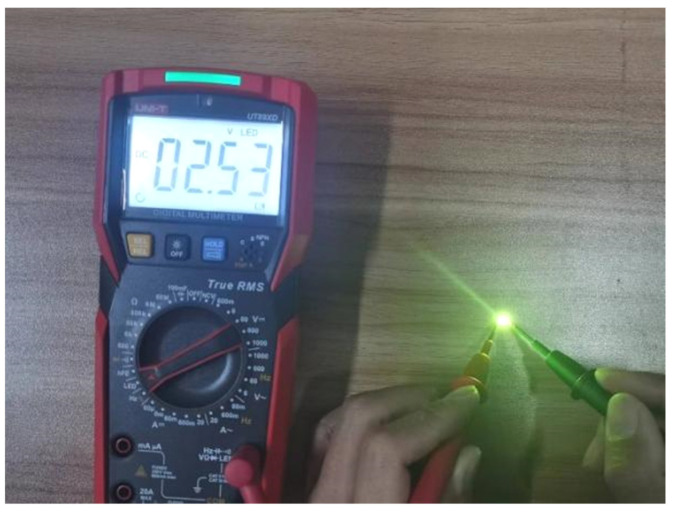
Sample failure inspection.

The cold/hot cycling test is mainly to assess the adaptability of the product to the sharp changes in the surrounding environment, which is an essential test in the routine test of the product batch production stage. The test samples were prepared with the three kinds of PCC bonding wires under their optimal bonding parameters, and the test samples were plastic-encapsulated with a DJ-XYZ300 fully automatic dispensing machine. Then, the test was carried out in the GDW-500 high/low temperature test chamber, which was maintained at high temperature (100 °C) and low temperature (−100 °C) for 5 min respectively, and then maintained at room temperature for 3 min, that is, 13 min as a cycle. The 50-, 100-, 150-, 200- and 250-cycle tests were carried out, respectively, and then a multimeter was used to inspect the samples and count the failed samples. As shown in Figure 2, an LED that can be powered on and lit up is considered a normal sample; otherwise, it is considered a failed sample.

## 3. Results and Discussion

### 3.1. Study of Pd80 Bonding Parameters

**Figure 3:**
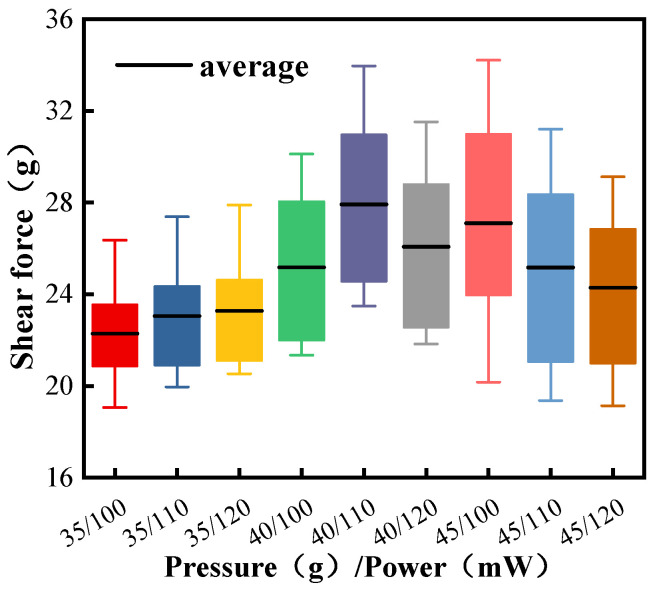
Shear force of Pd80 ball-bonded point under different bonding parameters.

Figure 3 shows the statistics of the shear force of the ball-bonded points of Pd80 under different bonding parameters. The bonding parameters of 40 g/110 mW (bonding pressure/ultrasonic power) and 45 g/100 mW have larger shear forces, and the average shear forces are 27.72 g and 27.10 g, respectively, but the former has more compact data distribution and significantly higher stability. Although the maximum value of the latter is relatively high, the data range is too wide and the stability is poor.

**Figure 4:**
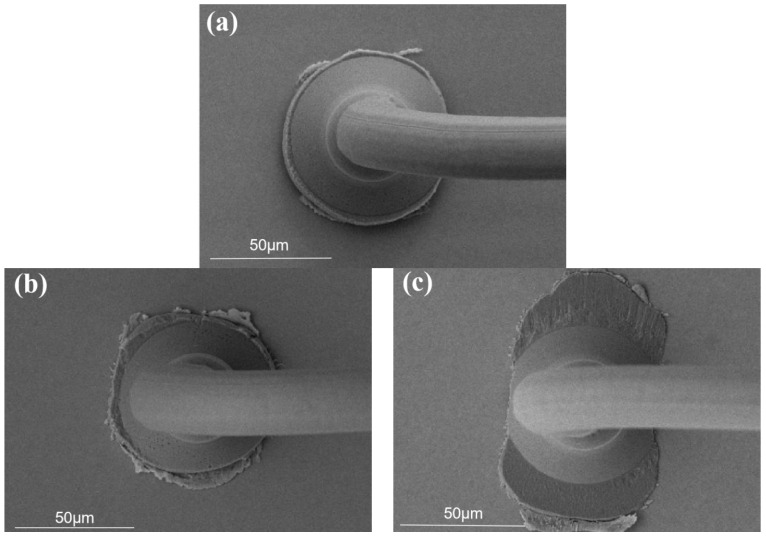

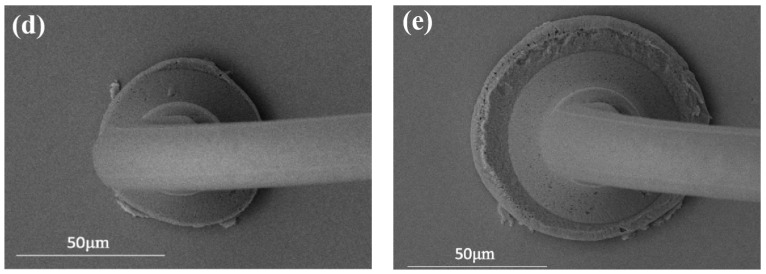
Morphology of Pd80 ball-bonded points under different bonding pressure/ultrasonic power. ((**a**) 40 g/110 mW; (**b**) 40 g/100 mW; (**c**) 40 g/120 mW; (**d**) 35 g/110 mW; (**e**) 45 g/110 mW).

When the bonding parameter is 40 g/110 mW, the morphology of the Pd80 ball-bonded point is shown in Figure 4a. The size and shape of the bonded point are regular, and have high bonding quality. The bonding parameter of 40 g/100 mW has a small bonded-point size compare to the pad, as shown in Figure 4b. When the bonding parameter is 40 g/120 mW, the pad surface is severely scratched and the pad is damaged, as shown in Figure 4c. The bonding parameter of 35 g/110 mW has a low degree of deformation of the bonded balls and a low bonding strength with respect to the pads, as shown in Figure 4d. When the bonding parameter is 45 g/110 mW, the pad is severely damaged, as shown in Figure 4e. Combined with Figure 3, when the bonding parameter is 40 g/110 mW, the shear force of the bonded point is the largest, and the morphology of the bonded point is regular, which is the optimal bonding parameter for the ball-bonded point.

**Figure 5:**
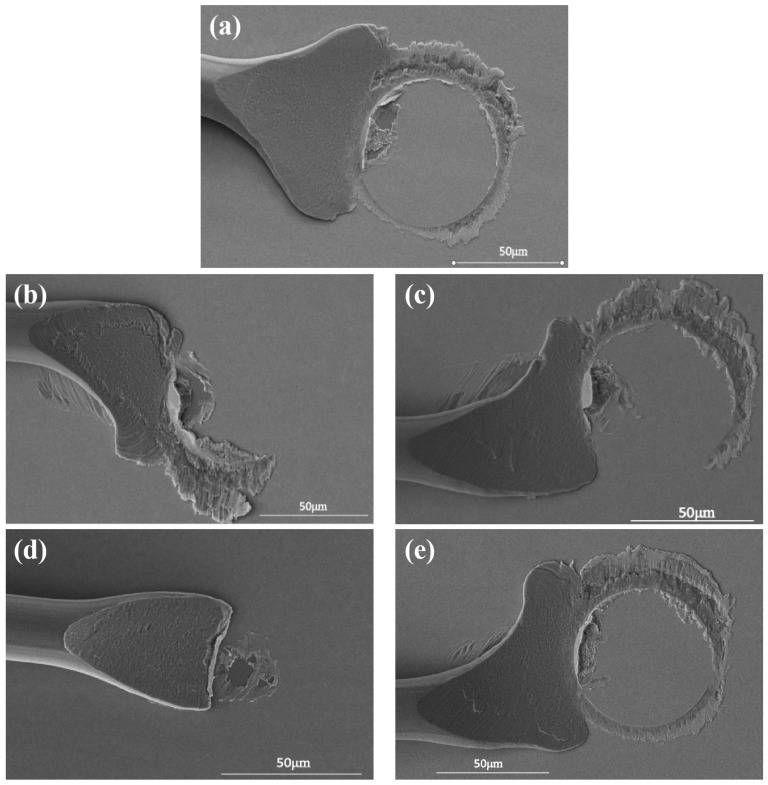
Morphology of Pd80 wedge-bonded points under different bonding pressure/ultrasonic power ((**a**) 45 g/105 mW; (**b**) 45 g/95 mW; (**c**) 45 g/115 mW; (**d**) 40 g/105 mW; (**e**) 50 g/105 mW).

The second bonded point is a wedge-bonded point, which is also called a fishtail, due to its shape. The combination of bonding parameters of the wedge-bonded point is shown in Table 1. Figure 5a shows the shape of the fishtail when the bonding parameter is 45 g/105 mW; the bonding wire undergoes sufficient plastic deformation during the bonding process, and the fishtail shape is regular and symmetric, with a complete ‘power ring’ imprint. The bonding parameter of 45 g/95 mW results in asymmetric and reduced width of the fishtail, an incomplete power ring, and low bond strength, as shown in Figure 5b. When the bonding parameter is 45 g/115 mW, there is an obvious ‘power ring’ mark, but the fishtail shape is irregular. The bonding wires vibrated on the surface of the silver pads with high amplitude due to excessive power, causing some degree of damage, as shown in Figure 5c. When the bonding parameter is 40 g/105 mW, the width of the fishtail is obviously small with the edge is warped, and there is no formation of the ‘power ring’. Because the pressure is too small, the bonding wire does not have sufficient plastic deformation to combine with the pads, resulting in low bond strength, as shown in Figure 5d. When the bonding parameter is 50 g/105 mW, the uneven force on the wedge weld leads to serious deformation of the fishtail, and the ‘power ring’ mark deepens, leading to pad breakage, as shown in Figure 5e. In conclusion, the optimal bonding parameter is 45 g/105 mW.

### 3.2. Study of Pd100 Bonding Parameters

**Figure 6:**
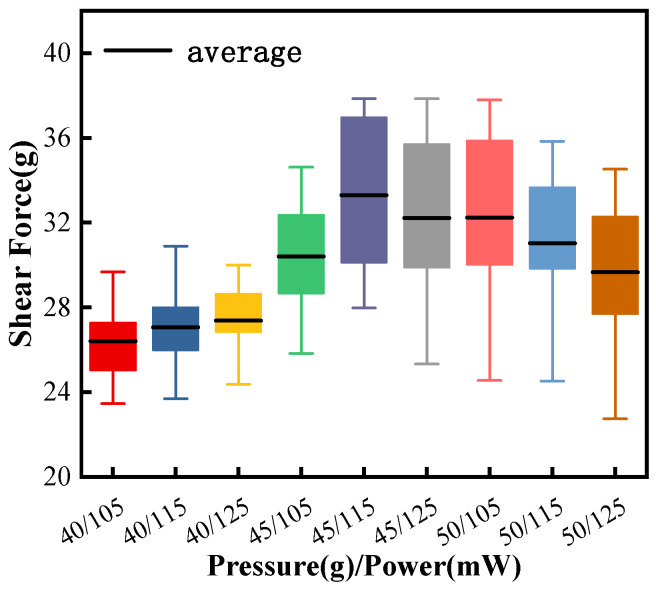
Shear of ball-bonded point with different combined bonding parameters.

As can be seen in Figure 6, a smaller bonding pressure corresponds to a lower shear-force value, indicating insufficient bonding strength. The ball-bonded point has the highest average value of shear and a lower dispersion when the bonding parameter is 45 g/115 mW. After increasing the bonding pressure or ultrasonic power, the shear value starts to decrease, indicating that the pads have been damaged to some extent, so the bonding parameter of 45 g/115 mW is more effective.

**Figure 7:**
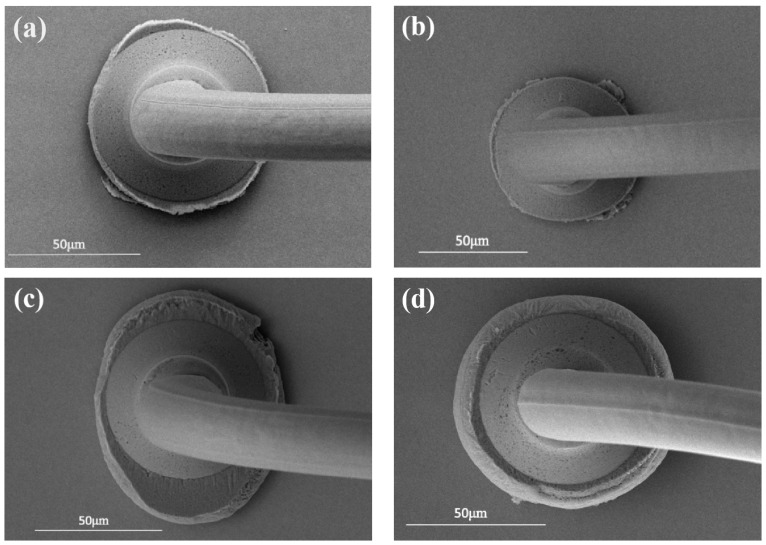
Morphology of Pd100 ball joints under different bonding pressures/ultrasonic power ((**a**) 45 g/115 mW; (**b**) 45 g/105 mW; (**c**) 45 g/125 mW; (**d**) 50 g/115 mW).

When the bonding parameter is 45/115 mW, the bonded point has regular shape, high roundness, and a large contact area with the pad, as shown in Figure 7a. The roundness of the bonded point is poor when the bonding parameter is 45 g/105 mW, and the contact area with the pad is small, so the bonding strength is low, as shown in Figure 7b. When the bonding parameter is 45 g/125 mW, the vibration amplitude of the bonded point on the surface of the pad increases due to the excessive power, resulting in serious extrusion of the silver pad and poor bonding quality, as shown in Figure 7c. The bonding parameter of 40 g/115 mW results in a small deformation of the bonded ball and does not produce a high-strength connection with the pad. When the bonding parameter is 50 g/115 mW, the silver pad at the edge of the ball-bonded point is damaged, due to the large amount of overflow from the pressure, as shown in Figure 7d. Combined with Figure 6, the bonding parameter 45 g/115 mW results in a more regular weld-joint morphology and high bond strength, which is the optimal bonding parameter.

**Figure 8:**
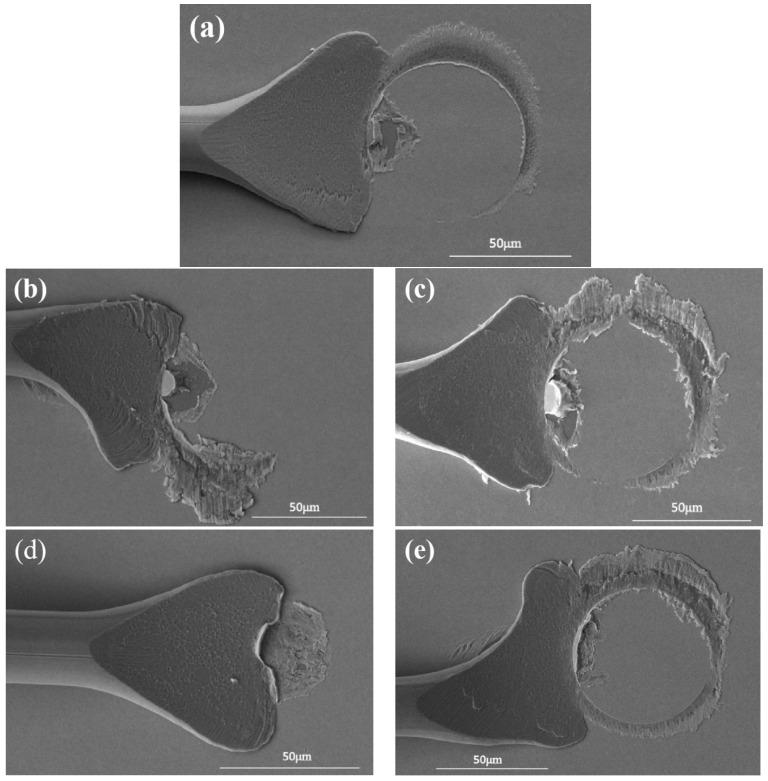
Morphology of Pd100 wedge-bonded points under different bonding pressures/ultrasonic power ((**a**) 50 g/110 mW; (**b**) 50 g/100 mW; (**c**) 50 g/120 mW; (**d**) 45 g/110 mW; (**e**) 55 g/110 mW).

The wedge-bonded point-bonding parameter combinations are shown in Table 2. Figure 8 shows the morphology of the wedge-bonded points of Pd100. When the bonding parameter is 50 g/110 mW, the shape of the bonded point is regular, and there is a ‘power ring’ mark without damaging the pad. The fishtail and pad contact area is larger so the bonding strength is high, as shown in Figure 8a. When the bonding parameter is 50 g/100 mW, there is no ‘power ring’ generated at the end of the fishtail, because the bonded point does not get enough energy and the plastic deformation is not sufficient. The fishtail size is small and the surface is not smooth, and the contact area with the pad is small, so it cannot form an effective connection and leads to low bond strength, as shown in Figure 8b. When the bonding parameter is 50 g/120 mW, the Ag spatters seriously around the ‘power ring’ and the fishtail shape is irregular, and the bonding pad is damaged, resulting in poor bonding quality, as shown in Figure 8c. When the bonding parameter is 45 g/110 mW, the wedge-bonded point is not completely plastic-deformed, and the fishtail edge cocked, due to the pulling action of the wire tail in the process of wire breakage. The fishtail is not tightly connected to the pad, as in Figure 8d; when the bonding parameter was 55 g/110 mW, the fishtail was asymmetric and shifted, which also caused Ag splatter on the pad surface. The damaged pad resulted in poor bonding quality, as shown in Figure 8e. In conclusion, the wedge-bonded point has regular morphology and high bonding quality at a bonding parameter of 50 g/110 mW, which is the best bonding parameter for Pd100.

### 3.3. Study of Pd120 Bonding Parameters

**Figure 9:**
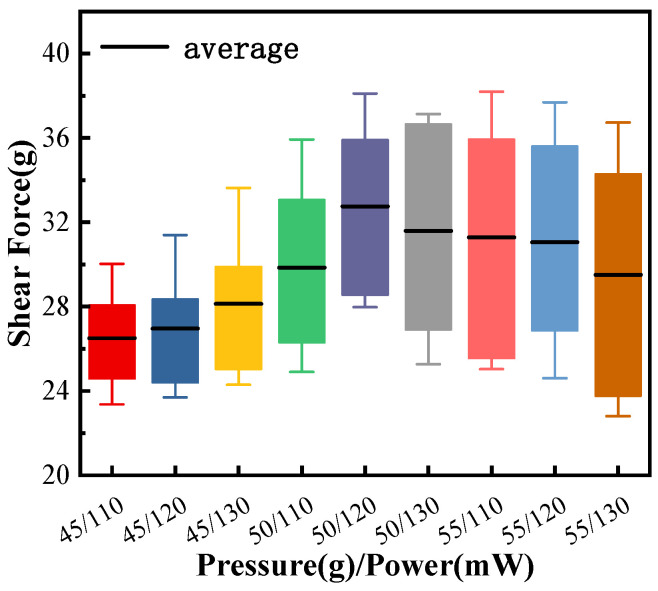
Shear of ball-bonded points with different combined bonding parameters.

Figure 9 is the statistics of shear-force data measured at the ball-bonded point. The shear-force average value of the bonding parameter is the largest at 50 g/120 mW. Although the maximum value is not the largest in all data, the data structure is compact and the average value is the highest, which proves that the bonding quality is high and stable.

**Figure 10:**
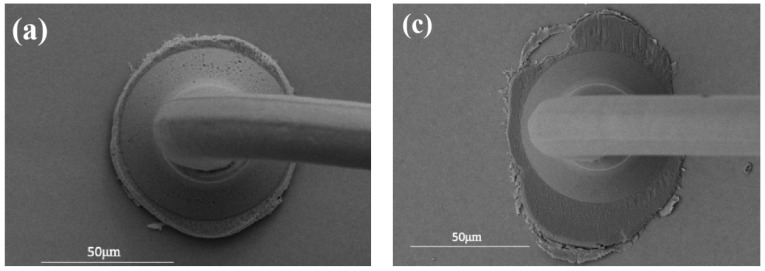

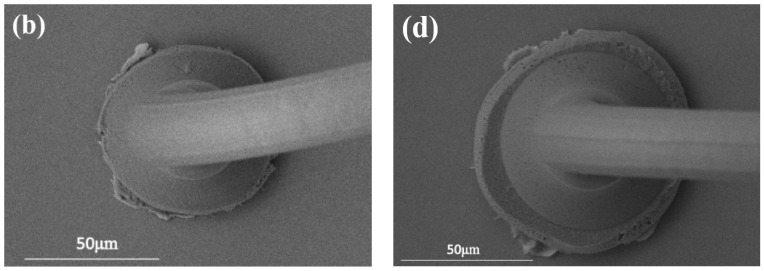
Morphology of Pd120 ball-bonded points under different bonding pressures/ultrasonic power ((**a**) 50 g/120 mW; (**b**) 50 g/110 mW; (**c**) 50 g/130 mW; (**d**) 55 g/120 mW).

Figure 10a shows the morphology of the ball-bonded point when the bonding parameter is 50 g/120 mW. The bonded point has a high degree of roundness and moderate diameter, and the contact area with the pads is large, so the bonding strength is high. When the bonding parameter is 50 g/110 mW, a Ag squeeze occurs in some areas at the edge of the bonded point, indicating that the reciprocating friction movement between the ball-bonded point and the pads is not sufficient during the bonding process, so a strong and effective connection is not formed, as shown in Figure 10b. When the bonding parameter is 50 g/130 mW, serious pad scratch occurs on both sides of the ball-bonded point, resulting in poor bonding quality and low strength. The main reason is that the excessive power causes the bonding ball-bonded points to move violently, resulting in scratches on the surface of the pads, as shown in Figure 10c. When the bonding parameter is 45 g/120 mW, the deformation of the bonded ball is insufficient, so there is no ‘Ag squeeze’ phenomenon. The small diameter of the bonded ball and the small contact area with the pad lead to low bond strength. When the bonding pressure is 55 g/120 mW, the phenomenon of silver splashing around the bonded point is obvious, as shown in Figure 10d. In conclusion, when the bonding parameter is 50 g/120 mW, the ball-bonded points have a regular morphology and high bonding quality, which is the optimal bonding parameter for the ball-bonded point.

**Figure 11:**
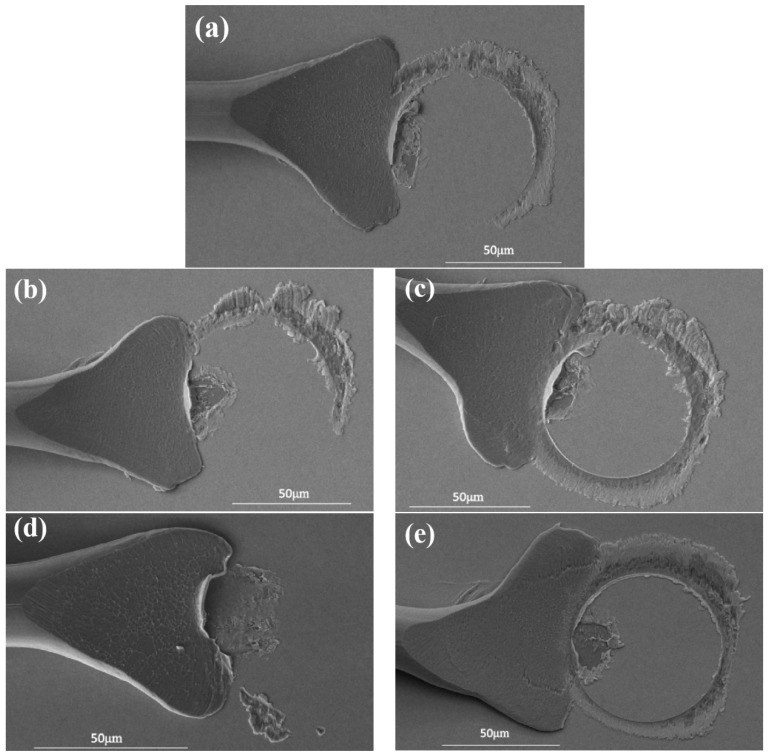
Morphology of Pd120 wedge-bonded points under different bonding pressures/ultrasonic power ((**a**) 55 g/115 mW; (**b**) 55 g/105 mW; (**c**) 55 g/125 mW; (**d**) 50 g/115 mW; (**e**) 60 g/115 mW).

Table 3 shows the combination of wedge-bonded point bonding parameters. Figure 11a shows the morphology of the Pd120 wedge-bonded point when the bonding parameter is 55 g/115 mW. At this time, the surface of the fishtail is smooth and symmetrical in shape, accompanied by a ‘power ring’ mark, with a large contact area with the pad and high bonding strength, meeting the requirements for use. When the bonding parameter is 55 g/105 mW, the width of the fishtail is narrow, and equivalent to only a half of the portion of the ‘power ring’ marks. This is mainly because the power is too small, and the bonding wire obtains less energy and not fully plastic deformation, so the fishtail and pad contact area is small, resulting in low strength of the connection, as shown in Figure 11b. When the bonding parameter is 55 g/125 mW, the width of the fishtail is moderate and a clear and complete ‘power ring’ mark appears at the end. However, the Ag splatter around the power ring is severe and the pad is damaged, resulting in reduced reliability, as shown in Figure 11c. When the bonding parameter is 50 g/125 mW, the fishtail is narrow and no ‘power ring’ is formed at the end. This is mainly due to the small pressure causing less deformation of the bonding wire, so the connection strength between the fishtail and the pad is low. In addition, due to the influence of the wire tail during wire breakage, the end of the fishtail is raised, as shown in Figure 11d. When the bonding parameter is 60 g/125 mW, the fishtail morphology is asymmetric, and the power ring imprint is too deep, causing damage to the pads and affecting the device life, as shown in Figure 11e. In summary, when the bonding parameter is 55 g/115 mW, the wedge-bonded points have regular shape and high strength, which is the optimal bonding parameter.

### 3.4. Effect of Pd-Layer Thickness on Bonding Reliability of Cu-Based Bonding Wire in High-Temperature Environment

**Figure 12:**
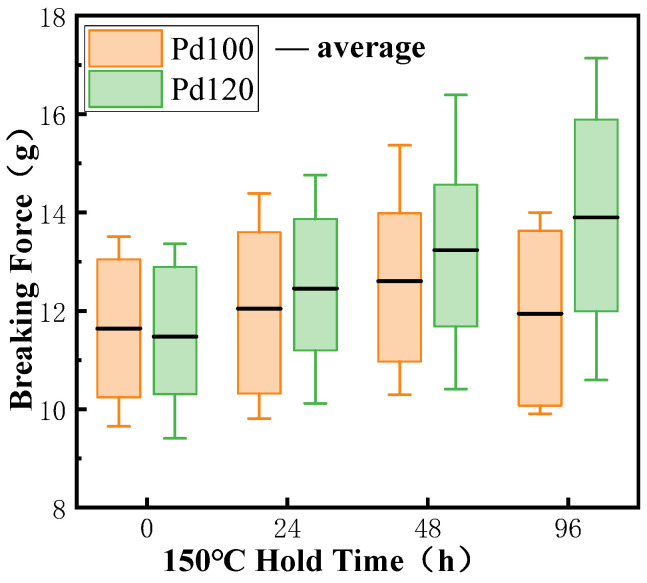
Tensile force of bonded point under different high-temperature storage times.

As shown in Figure 12, the average tensile value of Pd100 and Pd120 in the original state is around 11.5 g, while the average tensile value of Pd100 is slightly higher than that of Pd120. As Pd is absent at the bond interface in the as-bonded state, it does not affect bonding strength significantly [32]. With the increase of holding time during heating, the continuous high-temperature environment promoted the growth of Cu-Ag-IMCs, which improved the bonding strength. The mean value of the tensile force of Pd100 peaked at 12.6 g when stored at high temperature for 48 h. At 96 h, the mean value of the tensile force of Pd120 was 13.90 g and the maximum value of the tensile force was 17.14 g, while the maximum value of the tensile force of Pd100 decreased to 14.69 g, with an average value of 11.94 g. Because the prolonged high-temperature environment caused excessive IMC growth at the bonding interface of Pd100, the increase in Kirkendall holes reduced the connection strength. In contrast, Pd120 has a sustained increase in tensile strength due to the stronger hindering effect of Pd on Cu atoms, slower growth of IMCs, and formation of fewer holes.

**Figure 13:**
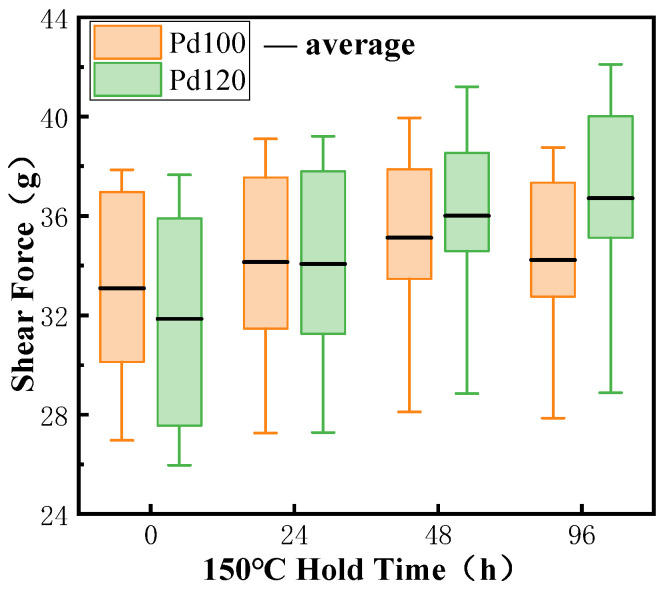
Shear force of ball-bonded point under different high-temperature storage times.

It can be seen from Figure 13 that the average value of Pd100 shear force in the original state is slightly larger than Pd120. This is because Pd does not participate in the formation of IMCs, and plays a role in blocking the diffusion of Cu and Ag atoms at the bonding interface and slowing down the growth of IMCs. Pd100 has a thinner plating layer and generates more IMCs quickly during the bonding process, so the bond point has a higher strength.

However, after the high-temperature storage test, the Pd atoms at the bonding interface will move to the periphery of the bonding region, the Cu and Ag atoms penetrate and diffuse into each other, and the IMCs at the bonding interface will gradually grow to form a higher-strength connection, so that both types of bonded-ball-point shear are improved. When stored at high temperatures for 96 h, the Pd100 ball-bonded-point shear starts to decrease because IMCs is sensitive to high temperatures and grows at a fast rate in high-temperature environments, which results in the creation of Kirkendall voids and a reduction in bonding strength. The shear of Pd120 continues to increase because the larger thickness of the Pd coating hinders and slows down the growth of IMCs, and Kirkendall voids have not yet formed. Its average value is 36.01 g, while the shear force of Pd100 is 34.23 g. It can be seen that PCC with a thicker Pd layer has higher bonding strength and reliability after high-temperature storage tests. At the same time, this also provides a basis for the coating thickness of bonding wires, which is best at not less than 0.47% of the wire diameter.

**Figure 14:**
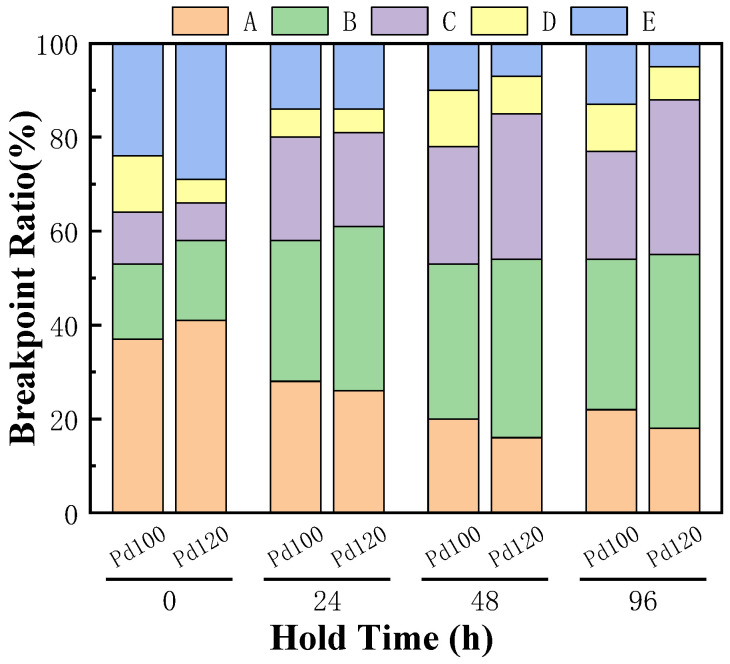
Breakpoint position proportion under different high-temperature storage times.

**Figure 15:**
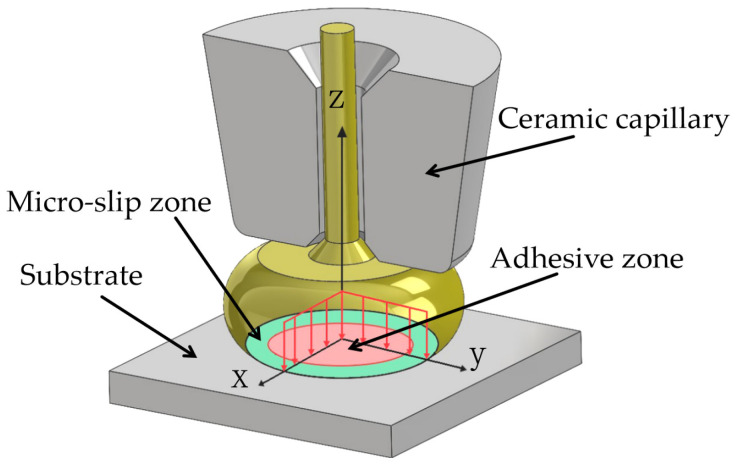
Partition of bonding interface.

Figure 14 shows the statistical diagram of the breakpoint positions of Pd100 and Pd120 after tensile testing under their respective optimal bonding parameters; 100 samples were tested for each parameter group. In the original state, the breakpoints of the two are mainly located at points A and E. Because the bonding points in these two areas have just formed and there are fewer IMCs, the strength of the bonding points is slightly lower than that of the bonding wire itself. Moreover, the high stress and strain-energy storage at the initial formation of the bonding point leads to poor stability, so these two positions are relatively weak compared to other parts, resulting in a higher proportion of fracture at points A and E. In addition, the contact surface between the ball-bonded point and the substrate can be divided into adhesive zone and micro-slip zone, as shown in Figure 15. The bonding strength is mainly determined by the IMCs generated by the micro-slip zone. The micro-slip zone of the ball-bonded point is slightly smaller than that of the wedge-bonded point, so the failure rate of point A is slightly higher than that of point E. On the other hand, the thicker Pd thickness hinders the formation and growth of IMCs for the bonding process, so the percentage of points A and E in the fracture location of Pd120 is higher than that of Pd100.

With the prolongation of the high-temperature storage test time, the Pd atoms move vigorously at the bonding interface and aggregate at the periphery, the IMCs grow gradually, and the bonding-point strength increases. Therefore, the percentage of fracture positions at points A and E gradually decreases, and the percentage of fracture positions at points B and C begins to increase (point C is the desired fracture position, because a fracture at C means a higher bond strength). When stored at high temperature for 48 h, the tensile breakpoints of the two kinds of samples are mainly concentrated at point B and C, and the proportions of the two fracture positions in Pd100 and Pd120 is 58% and 69%, respectively, which indicates that Pd can effectively slow down the formation and growth of harmful IMCs. When stored at high temperature for 96 h, the tension breakpoints of the two kinds of samples are still mainly at points B and C, but the proportion of the breakpoints A and E of Pd100 increases slightly. Because the thickness and area of IMCs further increase, the Kirkendall voids at the bonding interface also gradually increase, resulting in the bonding-point strength beginning to decrease. The proportion of the C point in Pd120 continues to increase, and its bonding strength continues to improve, which also proves that its bonded-point reliability is higher. It can be seen that the proportion of C points is positively correlated with the Pd thickness of the bonding wire; that is, the thicker the Pd layer, the higher the bonding strength, and the longer the service life of the device.

### 3.5. Effect of Pd-Layer Thickness on Bonding Reliability of Cu-Based Bonding Wires in Cold/Hot-Cycle Test

Pd80, Pd100 and Pd120 were used to make bonding samples, and then 100 samples were selected from each type for a cold/hot-cycle test. In the test, the thermal expansion, cold contraction and alternating stress of the sample caused by the temperature difference may lead to material cracking, poor contact and other phenomena, so its failure rate can reflect the reliability of the sample.

**Figure 16:**
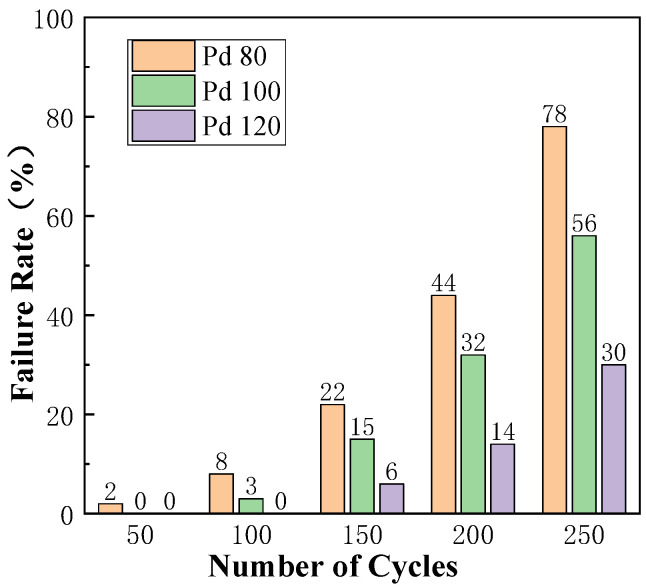
Sample failure rate under different cold/hot cycles.

It can be seen from Figure 16 that only Pd80 showed a failure condition when the cold/hot cycles occur 50 times, and only Pd120 did not fail after 100 times of cold and hot cycling. The failure rates of Pd80, Pd100, and Pd120 were 78%, 56%, and 30%, respectively, when there were 250 cold/hot cycles.

The larger the thickness of the Pd layer of PCC, the lower the failure rate of the sample, which indicates that the larger thickness of the Pd layer can improve bonding reliability. The main reasons are as follows: (1) with the increase in the thickness of the Pd layer, more Pd atoms exist at the bonding interface during the bonding process. Pd atoms do not participate in the formation of Cu Ag IMCs at the bonding interface, block the diffusion of Cu and Ag atoms, and slow down the growth rate of the IMCs. Therefore, the thicker the Pd layer, the thinner its IMC. Under the same number of cold/hot shocks, the influence of thermal expansion and cold contraction is relatively small, and its failure rate is relatively low; and (2) the thicker the Pd layer is, the more isolation layers it provides, which hinders the migration of grain boundaries and improves the recrystallization temperature of Cu-based bonding wires, thus shortening the heat-affected zone at the neck of the ball-bonded point. The internal stress of the neck is reduced, and the internal texture is uniform, which enhances the ability to resist deformation.

## 4. Conclusions

(1) For the ball-bonded point, when the bonding pressure and ultrasonic power are too small, the plastic deformation of the bonding ball is insufficient, the diameter is small, the contact area with the pad is small, the bonding quality is poor, and the strength is low. When the bonding pressure and ultrasonic power are too large, there will be a large range of Ag spatter and ‘scratch’ on the surface of the pad, which will damage the pad and affect the bonding quality and strength of the bonded point.

(2) For the wedge-bonded point, when the bonding pressure and ultrasonic power are too small, the width of the fishtail will be narrowed and the tail end will be cocked, resulting in false bonding. When the bonding pressure and ultrasonic power are too large, it will cause stress concentration, and the pad will appear to have an ‘internal injury’, which increases the risk of failure.

(3) A short-term high-temperature environment can increase the PCC tensile/shear force and bonding strength; with the extension of high-temperature storage time, the push–pull value of Cu-based bonding wire with small Pd-layer thickness will first reach the peak value, and then begin to decline, while the tensile/shear force of Cu-based bonding wire with large Pd-layer thickness will continue to rise.

(4) In a high-temperature environment, the greater the thickness of the Pd layer of PCC, the greater the proportion of the fracture point C, indicating that the bonding strength is higher; in the cold/hot cycle test with sudden temperature change, the failure rate of PCC with large Pd-layer thickness is the lowest, which indicates that the greater the thickness of the Pd layer, the stronger its ability to resist changes in the external environment, and further indicates its high stability and reliability.

## Figures and Tables

**Table 1:** Combination of bonding parameters of Pd80 wedge-bonded point.

Number	Bonding Pressure/g	Ultrasonic Power/mW
1	40	95
2	40	105
3	40	115
4	45	95
5	45	105
6	45	115
7	50	95
8	50	105
9	50	115

**Table 2:** Combination of bonding parameters of Pd100 wedge-bonded points.

Number	Bonding Pressure/g	Ultrasonic Power/mW
1	45	100
2	45	110
3	45	120
4	50	100
5	50	110
6	50	120
7	55	100
8	55	110
9	55	120

**Table 3:** Combination of bonding parameters of Pd120 wedge-bonded points.

Number	Bonding Pressure/g	Ultrasonic Power/mW
1	50	105
2	50	115
3	50	125
4	55	105
5	55	115
6	55	125
7	60	105
8	60	115
9	60	125

## Data Availability

The data used to support the findings of this study are included within the article.
